# An automatic identification system for citrus greening disease (Huanglongbing) using a YOLO convolutional neural network

**DOI:** 10.3389/fpls.2022.1002606

**Published:** 2022-12-20

**Authors:** Rong-Zhou Qiu, Shao-Ping Chen, Mei-Xiang Chi, Rong-Bo Wang, Ting Huang, Guo-Cheng Fan, Jian Zhao, Qi-Yong Weng

**Affiliations:** Fujian Key Laboratory for Monitoring and Integrated Management of Crop Pests, Institute of Plant Protection, Fujian Academy of Agricultural Sciences, Fuzhou, China

**Keywords:** citrus greening, Huanglongbing, automated identification, deep learning, convolutional neural networks

## Abstract

Huanglongbing (HLB), or citrus greening disease, has complex and variable symptoms, making its diagnosis almost entirely reliant on subjective experience, which results in a low diagnosis efficiency. To overcome this problem, we constructed and validated a deep learning (DL)-based method for detecting citrus HLB using YOLOv5l from digital images. Three models (Yolov5l-HLB1, Yolov5l-HLB2, and Yolov5l-HLB3) were developed using images of healthy and symptomatic citrus leaves acquired under a range of imaging conditions. The micro F1-scores of the Yolov5l-HLB2 model (85.19%) recognising five HLB symptoms (blotchy mottling, “red-nose” fruits, zinc-deficiency, vein yellowing, and uniform yellowing) in the images were higher than those of the other two models. The generalisation performance of Yolov5l-HLB2 was tested using test set images acquired under two photographic conditions (conditions B and C) that were different from that of the model training set condition (condition A). The results suggested that this model performed well at recognising the five HLB symptom images acquired under both conditions B and C, and yielded a micro F1-score of 84.64% and 85.84%, respectively. In addition, the detection performance of the Yolov5l-HLB2 model was better for experienced users than for inexperienced users. The PCR-positive rate of *Candidatus* Liberibacter asiaticus (CLas) detection (the causative pathogen for HLB) in the samples with five HLB symptoms as classified using the Yolov5l-HLB2 model was also compared with manual classification by experts. This indicated that the model can be employed as a preliminary screening tool before the collection of field samples for subsequent PCR testing. We also developed the ‘HLBdetector’ app using the Yolov5l-HLB2 model, which allows farmers to complete HLB detection in seconds with only a mobile phone terminal and without expert guidance. Overall, we successfully constructed a reliable automatic HLB identification model and developed the user-friendly ‘HLBdetector’ app, facilitating the prevention and timely control of HLB transmission in citrus orchards.

## 1 Introduction

Citrus is the most widely cultivated fruit tree species in southern China. Citrus Huanglongbing (HLB), or citrus greening disease, is the most devastating disease for the citrus-producing industry associated with the pathogen *Candidatus* liberibacter asiaticus (CLas). Affected plants are small, deformed, produce green or “red nose” fruits, and are likely to develop other problems including tree vigour decline, fruit yield reduction, and quality degradation. While there is no treatment for the disease, HLB symptoms can be alleviated by spraying additional foliar mineral nutrients and plant-growth regulators including plant hormones, such as gibberellin, and synthetic plant hormone derivatives (Bassanezi et al., 2021; Ma et al., 2022); however, this does not reduce the incidence of HLB in orchards, and can even increase the risk of transmission.

To ensure healthy citrus orchards, strict vector control through insecticide spraying and the removal of diseased plants in HLB-affected orchards remains the best long-term control measure (Bassanezi et al., 2021; Yuan et al., 2021). The key to the successful implementation of this measure is the early detection of HLB-affected plants. However, HLB-affected plants often have a variety of symptoms, such as blotchy mottling, uniform yellowing, zinc-deficiency, and “red nose” fruits, which can make HLB diagnosis difficult.

Morphological classification and diagnosis in the field and polymerase chain reaction (PCR) in a laboratory (Jagoueix et al., 1996) are the most commonly used methods for identifying HLB. Because the symptoms of HLB-affected plants are diverse, morphological classification and diagnosis in the field require extensive practical experience, background knowledge, and a basic understanding of the orchard being investigated. Diagnosis is, therefore, somewhat subjective, and the rate of misdiagnosis can exceed 30% (Futch et al., 2009). PCR testing is more reliable for diagnosis but requires highly skilled operators with specialised equipment, and involves a cumbersome and lengthy process, which reduced efficiency when detecting HLB-affected citrus plants (Li et al., 2007). Given that accurate field diagnosis of citrus HLB is an important skill for citrus producers, there is an urgent need for a rapid, reliable, and field-applicable testing method for early detection that will allow citrus producers to detect affected plants as early as possible.

Previous studies have employed various sensor techniques and simulation models to identify HLB-affected leaves, including thermal imaging (Sankaran et al., 2013), chlorophyll fluorescence spectroscopy (Weng et al., 2021), laser-induced fluorescence spectroscopy (Pereira et al., 2011), visible spectroscopy (Gómez-Flores et al., 2019), near-infrared spectroscopy (Sankaran and Ehsani, 2011), and hyperspectral sensors (Deng et al., 2019). While these studies show promising experimental results, these techniques currently have limited practical applications because they are relatively expensive (Picon et al., 2019).

Deep learning (DL) is a relatively new artificial intelligence technique that offers state-of-the-art modelling performance (LeCun et al., 2015). Among the range of DL methods available, Convolutional Neural Networks (CNNs) have shown excellent potential for the automatic extraction of visible features, and have been widely employed in agricultural applications including plant disease recognition (Ma et al., 2018; Sun et al., 2021; Yadav et al., 2021; Hua et al., 2022; Zhou et al., 2022), pest detection (Cheng et al., 2017; Roosjen et al., 2020; Hong et al., 2021; Dai et al., 2022), and fruit detection (Bresilla et al., 2019; Afonso et al., 2020; Lawal, 2021). Representative CNN algorithms include Region-based Convolution Neural Networks (R-CNN) (Ren et al., 2017), Fast R-CNN (Ren et al., 2017), Single Shot MultiBox Detector (SSD) (Liu et al., 2016), and You Only Look Once (YOLO) (Redmon et al., 2016). The YOLO series represents one-stage algorithms, which are more suited to practical applications than two-stage algorithms (such as Faster R-CNN) owing to their better balance between accuracy and speed. For instance, Lawal (2021) proposed improved YOLO-Tomato models for tomato detection under uneven environmental conditions, achieving higher detection accuracy and speed than Fast R-CNN.

Previous studies have showed that YOLO algorithms perform better than other two-stage algorithms in plant disease recognition. For instance, SSD, Faster R-CNN, and YOLO algorithms have been applied to detect tomato diseases and pests, with the YOLO algorithm providing superior detection accuracy and speed (Liu and Wang, 2020). Similarly, Wang et al. (2021) showed that the accuracy and speed of the YOLO framework was better when constructing models aimed at detecting tomato diseases and pests compared to those of Faster R-CNN, Mask R-CNN, and SSD, even with image occlusion and overlapping in the natural environment. Mathew and Mahesh (2022) also employed YOLOv5 to detect bacterial spot disease in bell pepper plants, achieving better accuracies and speeds than those obtained from previous versions of the YOLO algorithm. Although studies have shown that the YOLO framework holds great promise for plant disease recognition, it relies on large datasets and there are few open datasets available for plant diseases. Furthermore, sample collection in some studies is not always consistent with field conditions, which can lead to inaccuracies and limits wider application (Mohanty et al., 2016; Barbedo, 2019).

Here, we built a dataset without image augmentation containing 7,516 images, including images of healthy citrus leaves and fruits (1,413 images), five HLB symptoms (including blotchy mottling, “red-nose” fruits, zinc-deficiency, vein yellowing, and uniform yellowing; 3,017 images), and seven other citrus disease symptoms (including magnesium-deficiency, boron-deficiency, anthracnose, citrus greasy spot, citrus moss, sooty mould, and canker; 3,086 images). We then constructed citrus HLB detection models with different dataset combinations using YOLOv5l, and selected an optimal model for further testing using different test data obtained under different scenarios. In addition, we used the PCR-positive rate of CLas to examine the feasibility of using our model for the automated diagnosis of citrus HLB. Finally, we interfaced our model with an Android app that instantly detects citrus tree HLB infection in real-time. To our knowledge, our study is the first to employ YOLO for citrus HLB identification based on a primary image dataset without image augmentation.

## 2 Methods

### 2.1 Sample collection

**Figure 1:**
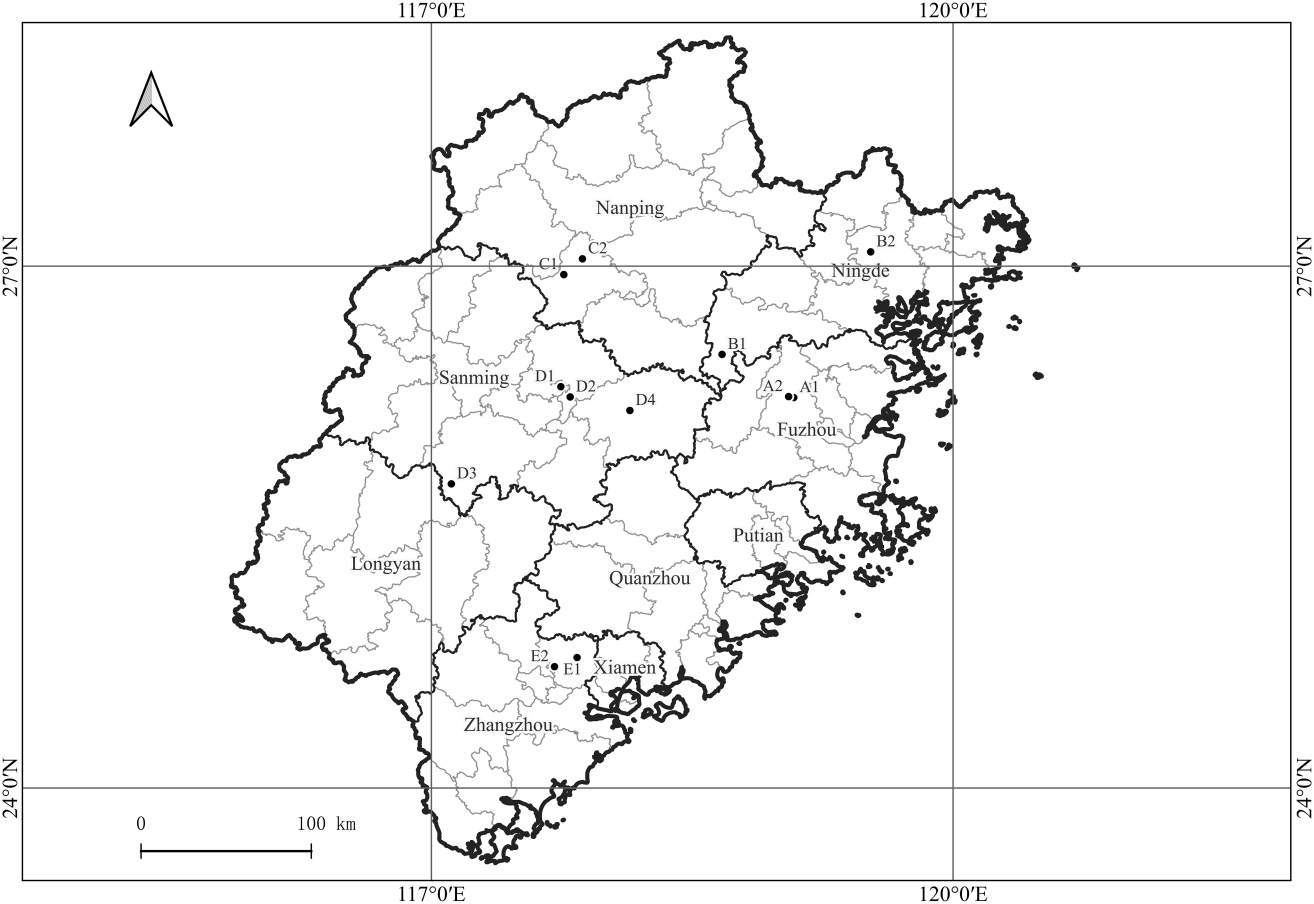
Geographical locations of citrus orchards in Fujian Province, China, from which plant images were acquired. A1, A2: Fuzhou (Navel orange, *Citrus sinensis* Osb. var. *brasiliensis* Tanaka); B1, B2: Ningde (Navel orange, *C. sinensis* Osb. var. *brasiliensis* Tanaka); C1: Nanping (Shatian pomelo, *C. grandis* var. *shatinyu* Hort); C2: Nanping (Ponkan, *C. reticulate* Blanc Ponkan); D1: Sanming (Shatangju mandarin, *C. reticulata* cv Shatangju; Orah); D2: Sanming (Satsuma mandarin, *C. unshiu* Marc.); D3: Sanming (Hongmeiren citrus hybrid); D4: Sanming (Ponkan, *C. reticulate* Blanc Ponkan); E1: Zhangzhou (Ponkan, *C. reticulate* Blanc Ponkan); E2: Zhangzhou (Ponkan, *C. reticulate* Blanc Ponkan; Tankan, *C. reticulata* var. *tankan*).

Samples were collected from 12 citrus orchards in Fuzhou, Ningde, Nanping, Sanming, and Zhangzhou cities in Fujian Province, China (**Figure 1**). The sampled species were the Ponkan (*Citrus reticulate* Blanc Ponkan), Tankan (*C. reticulata* var. *tankan*), Satsuma mandarin (*C. unshiu* Marc.), Orah mandarin, Hongmeiren citrus hybrid, Shatangju mandarin (*C. reticulata* cv Shatangju), Navel orange (*C. sinensis* Osb. var. *brasiliensis* Tanaka), and Shatian pomelo (*C. grandis* var. *shatinyu* Hort). Images of citrus plants with HLB, canker, citrus greasy spot, anthracnose, sooty mould, magnesium-deficiency, boron-deficiency, and citrus moss were acquired along with images of healthy citrus plants. The leaves used for the image acquisition were intact and naturally expanded, with corresponding intact fruits with a clearly visible pedicel base.

A total of 7,516 disease and healthy images were captured at a distance of 50–150 mm from the sample and under different conditions by five experimenters using the following mobile phones and digital cameras: a Hornor Play4T Promobile phone (HUAWEI Technologies Co., Ltd, Shenzhen, China); an MI 9 mobile phone (XIAOMI Technologies Co., Ltd., Beijing, China); an MI 5X mobile phone (XIAOMI Technologies Co., Ltd, Beijing, China); an iPhone 12 (Apple Technologies Co., Ltd., Silicon Valley, United States); and a Sony-RX100 digital camera (Sony Technologies Co., Ltd, Tokyo, Japan). The weather at the time of sampling was either sunny, cloudy, or rainy. Since the images were taken between 8:00 am and 06:00 pm, the final dataset contained images acquired under different light intensities, which ensured the adaptability of the method to different illumination conditions. To increase sample diversity, some samples were also photographed indoors with white or black plates as a background.

As the images were captured at variable pixel resolutions (3,000 × 4,000; 2,250 × 4,000; or 3,648 × 5,472 pixels), the captured original images were uniformly processed in Photoshop to JPG format with an image resolution of 72 pixels/inch while proportionally down-scaling their size to 640 × m pixels (m ≤ 640 pixels). To improve the efficiency of sample labelling, the custom-made sample-labelling software ‘HyperSpider LabelTool’ was used to indicate the HLB-affected and unaffected leaves and fruits with bounding boxes along with annotations of plant coordinates and health status categories. For a given leaf or fruit, the bounding box was minimised to cover only the target so that the number of background pixels inside the box was reduced to the allowable minimum. The annotation files were stored in TXT format with the same names as the corresponding images.

### 2.2 Image dataset construction

Sample symptoms were classified by experts into the following five major categories and 14 subcategories (S1–S14) based on differences in leaf and fruit symptoms (**Table 1**):

**Table 1:** Symptom categories, varieties, and sample sizes of citrus image database acquired under different conditions.

Major categories of symptom	Subcategories of symptom	Citrus varieties	Acquisition condition A			Acquisition condition B	Acquisition condition C
			Training sets	Validation sets	Test sets	Test sets	Test sets
Healthy (Dataset A)	Healthy fruit (S1)	Ponkan, Orah	537	60	57	43	43
	Healthy leaf (S2)	Ponkan, Orah	471	60	56	43	43
Typical HLB symptoms (Dataset B)	Blotchy mottling (S3)	Ponkan, Tankan, Shatian pomelo	586	70	67	43	43
	“Red-nose” fruit (S4)	Ponkan, Tankan, Navel orange	208	26	26	43	43
Suspected HLB symptoms (Dataset C)	Zinc-deficiency (S5)	Ponkan, Tankan, Hongmeiren	622	80	76	43	43
	Vein-yellowing (S6)	Ponkan, Tankan, Navel orange	391	47	44	43	43
	Uniform yellowing (S7)	Ponkan, Tankan, Orah	275	35	34	43	43
HLB-like symptoms (Dataset D)	Magnesium-deficiency (S8)	Ponkan, Satsuma mandarin	262	33	32	43	43
	Boron-deficiency (S9)	Navel orange	254	32	32	43	43
	Anthracnose (S10)	Ponkan, Shatangju mandarin	286	36	36	43	43
	Citrus greasy spot (S11)	Ponkan, Satsuma mandarin	202	27	27	43	43
	Citrus moss (S12)	Ponkan, Satsuma mandarin	361	45	44	43	43
HLB-irrelevant symptoms (Dataset E)	Sooty mould (S13)	Ponkan, Satsuma mandarin	327	42	36	43	43
	Canker (S14)	Orah, Navel orange	298	37	35	43	43
Total			5080		602	602	602

**Figure 2:**
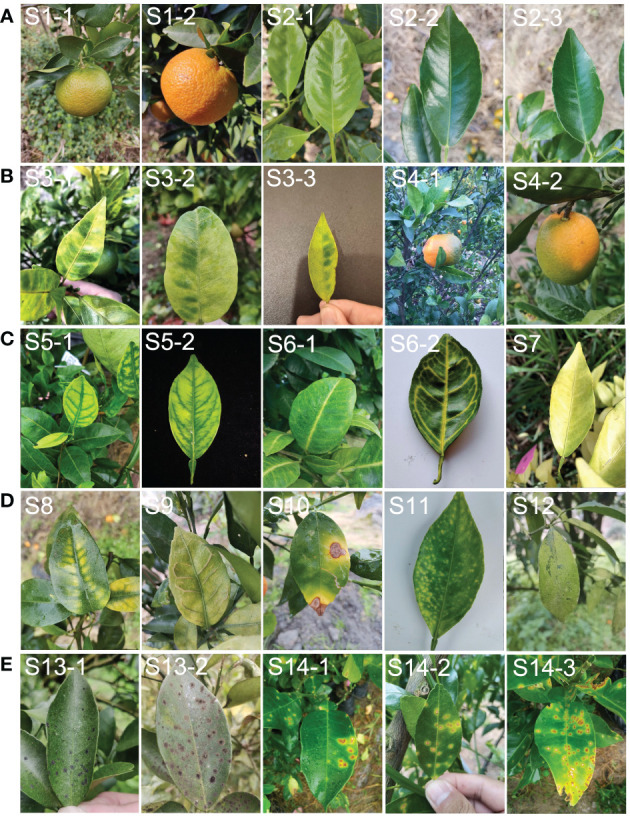
Example of leaf and fruit images with different symptoms used in the study. **(A)**, representative images classified as dataset ‘A’ (healthy). **(B)**, representative images classified as dataset ‘B’ (typical HLB symptoms). **(C)**, representative images classified as dataset ‘C’ (suspected HLB symptoms). **(D)**, representative images classified as dataset ‘D’ (HLB-like symptoms). **(E)**, representative images classified as dataset ‘E’ (HLB-irrelevant symptoms). S1, healthy fruit; S2, healthy leaf; S3, blotchy mottling; S4, “red-nose” fruit; S5, zinc-deficiency; S6, vein-yellowing; S7, uniform yellowing; S8, magnesium-deficiency; S9, boron-deficiency; S10, anthracnose; S11, citrus greasy spot; S12, citrus moss; S13, Sooty mould; S14, canker.

Dataset A: images of healthy fruits (S1) and leaves (S2) from healthy plants (**Figure 2A**). These images covered both new and old leaves as well as green fruits before colour change and mature fruits after colour change.

Dataset B: images of fruits and leaves from plants with one of two typical HLB symptoms, including blotchy mottling (S3) and “red-nose” fruits (S4) (**Figure 2B**). Blotchy mottling referred to uneven alternation between yellow and green colours, while “red-nose” fruits refer to fruits that are orange-red at the stalk end and cyan elsewhere.

Dataset C: images of leaves from plants with suspected HLB symptoms, covering three types of yellowing including zinc deficiency-induced yellowing (i.e., zinc deficiency) (S5), vein yellowing (S6), and uniform yellowing (S7) (**Figure 2C**). Uniform yellowing referred to the whole leaf turning yellow; zinc deficiency-caused yellowing referred to the veins turning a blue-green and the mesophylls turning yellow; and vein yellowing referred to the veins turning yellow and the mesophylls turning green or yellow with clear boundaries between the veins and mesophylls.

Dataset D: images of leaves in plants mainly affected by non-HLB diseases with the following HLB-like symptoms: magnesium deficiency (S8), boron deficiency (S9), anthracnose (S10), citrus greasy spot (S11), and citrus moss (S12) (**Figure 2D**). The magnesium deficiency symptoms included yellowing of the leaves in an inverted V-shape; the boron deficiency symptom was a swelling of the veins; the anthracnose symptom referred to a concentric ring-like pattern of black dots on the leaves; the citrus greasy spot symptom was the development of yellow patches or brown greasy spots on the leaves; and the citrus moss symptoms included green epiphytic chlorellas and moss in a fuzzy, lumpy, or irregular shape on the leaves.

Dataset E: images of leaves from plants affected by non-HLB diseases without HLB-like symptoms, such as sooty moulds (S13) and cankers (S14) (**Figure 2E**). The sooty mould symptoms included a black or dark-brown layer of fuzzy mould on the leaves, and the canker symptoms included lesions with volcano-shaped cracking in the centre.

To test the generalised performance of the final model, the sample images were also classified under the following acquisition conditions according to different photographic devices used and the environmental conditions at the time of acquisition (**Table 1**):

Acquisition condition A: a total of 6,312 images acquired using Huawei and Xiaomi mobile phones in the field or indoor against a background plate (either solid white or solid black), from which all training datasets as well as test sets T1–T3 were constructed (**Table 2**).

**Table 2:** Symptoms and sample sizes of test datasets acquired under different conditions.

Test datasets	Symptom	Number of images	Acquisition condition
T1	S (1–7)	360	A
T2	S (1–12)	531	A
T3	S (1–14)	602	A
T4	S (1–12)	516	B
T5	S (1–14)	602	B
T6	S (3–9)	301	B
T7	S (1–12)	516	C
T8	S (1–14)	602	C
T9	S (3–9)	301	C

S1, healthy fruit; S2, healthy leaf; S3, blotchy mottling; S4, “red-nose” fruit; S5, zinc-deficiency; S6, vein-yellowing; S7, uniform yellowing; S8, magnesium-deficiency; S9, boron-deficiency; S10, anthracnose; S11, citrus greasy spot; S12, citrus moss.

Acquisition condition B: a total of 602 images acquired in the field using a camera and an Apple iPhone (43 photos for each symptom subcategory), from which test sets T4–T6 were constructed (**Table 2**).

Acquisition condition C: the same leaf or fruit samples photographed under acquisition condition B but isolated from the plants and placed on a white background plate for secondary photography with a camera or mobile phone. A total of 602 images were collected (43 images for each symptom subcategory), from which test sets T7–T9 were constructed (**Table 2**).

The plants selected for photography, the time of image acquisition, and the photographers undertaking acquisition conditions B and C were different from those of acquisition condition A.

### 2.3 Experimental setup

**Figure 3:**
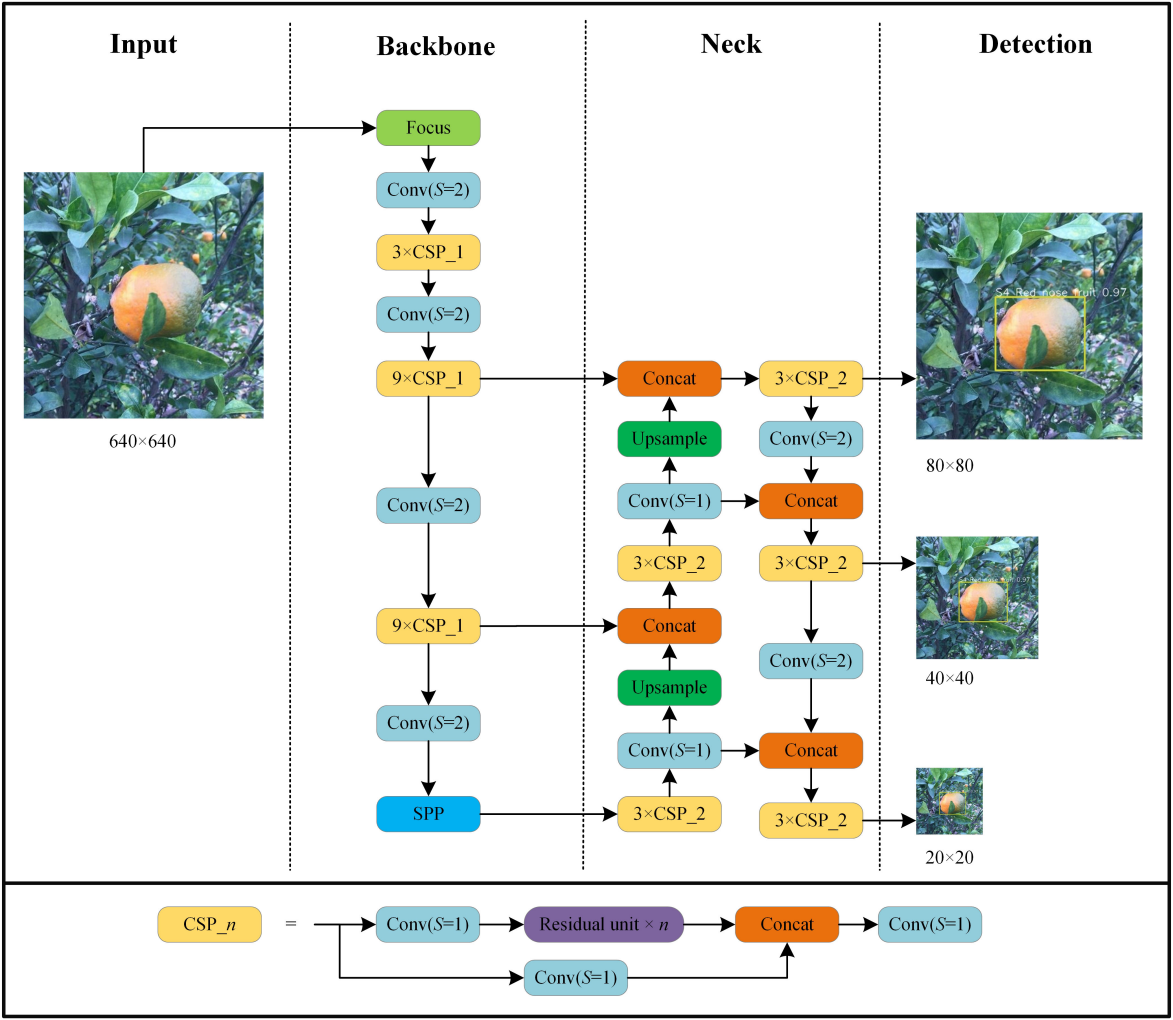
Architecture of YOLO v5l network used in this study. Focus is lossless down sampling. Conv (S=2) denotes convolution with a step size of 2 and a convolution kernel size of 3×3. Conv (S=1) uses a filter with a size of 1×1 and convolution with a step size of 1. CSP_n denotes a convolution module integrated with n residual units. SPP denotes spatial pyramid pooling. Concat is the feature map fusion operation. Upsample is the upsampling operation.

We used the YOLO v5l algorithm (Jocher et al., 2021) and its implementation in the Darknet library to create several HLB-detection models using the collected image datasets. The network structure of HLB detection based on YOLO v5l is shown in **Figure 3**. The parameters used to train the network included a base learning rate = 0.001; momentum = 0.937; weight decay = 0.0005; batch size = 20; and epoch = 200. All experiments were run using a computer with a GeForce RTX 3090 GPU.

**Figure 4:**
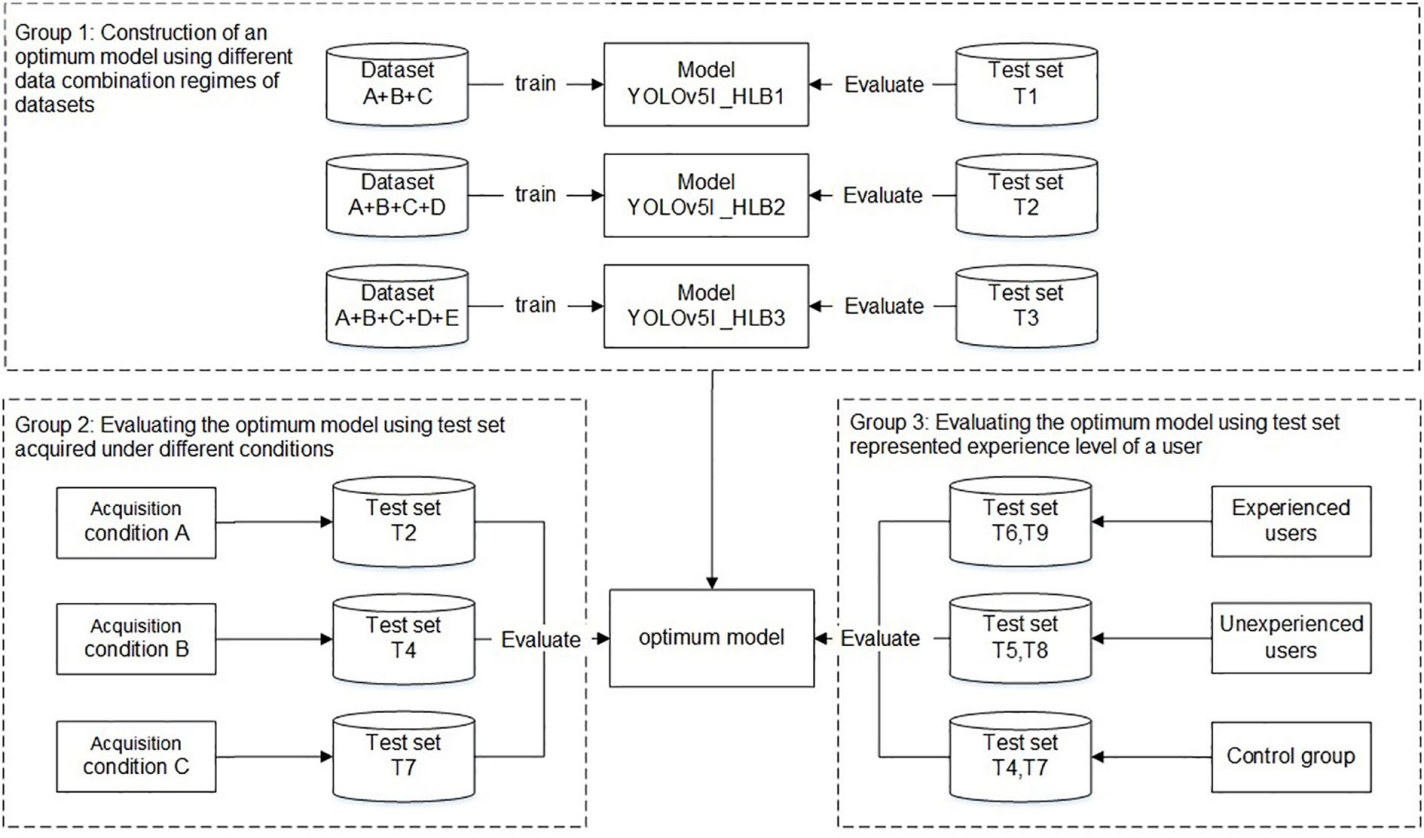
Workflow used to develop and evaluate a HLB-detection model based on the YOLO (You Only Look Once) image-detection system.

The experiments were divided into three main groups (**Figure 4**). The first group aimed to develop an optimal model using different data combination regimes. The YOLO v5l network was trained and validated using datasets A+B+C, datasets A+B+C+D, and datasets A+B+C+D+E, which yielded three HLB-detection network models, referred to as Yolov5l_HLB1, Yolov5l_HLB2, and Yolov5l_HLB3. To identify the optimal model, the three models were evaluated using the corresponding test sets T1, T2, and T3 (**Table 2**), respectively. In each case, the datasets captured under acquisition condition A were split using about 80% for training, 10% for validation, and 10% for testing (**Table 1**).

The second group focused on the influence of the test set on the recognition accuracy of the selected optimal model in the first group under different acquisition conditions, focusing on inconsistency in image acquisition conditions between the test samples and the training samples (Barbedo, 2016). Test sets T2, T4, and T7 (**Table 2**), which were captured under acquisition condition A, B, and C, respectively, but contained the same symptom subcategories (S1–S12) were used to validate the symptom-recognition accuracy of the optimal model selected in the first group. A confusion matrix was employed to assess the accuracy of the selected model for recognising all 14 symptoms captured under the different acquisition conditions. F1-scores were further employed to assess the accuracy of the selected model in recognising the five HLB symptoms (i.e., blotchy mottling, S3; “red-nose” fruits, S4; zinc deficiency, S5; vein yellowing, S6; and uniform yellowing, S7) captured under the different acquisition conditions.

The third group focused on confirming whether the knowledge or experience level of a user (i.e., the sample collector) impacted the model’s performance in practice. T6 and T9 (**Table 2**), which contained the same symptom subcategories), represented two types of image test sets acquired by experienced users under conditions B and C, respectively; T5 and T8 (**Table 2**), which contained the same symptom subcategories (S1–S14), represented two types of image test sets acquired by inexperienced users under conditions B and C, respectively; and, finally, T4 and T7 (**Table 2**) were employed as control sample images that were acquired under conditions B and C, respectively, and contained the same 12 symptom subcategories (S1–S12) as the training set.

### 2.4 Evaluation criteria

The constructed models were evaluated using different metrics including precision (*P*), recall (R), F1-scores (*F*1), and confusion matrices. A detailed explanation of these evaluation metrics is described by Hossin and Sulaiman (2015). The precision, recall and F1-scores are calculated as follows:


(1) $$Precision\;(P)=\frac{TP}{TP+FP}$$

(2) $$Recall\;(R)\;=\;\frac{TP}{TP+FN}$$

(3) $$F1-Scores\;(F1)=\frac{2PR}{P+R}$$

where *TP*, *FP* and *FN* represent the number of true positive cases, false positive cases and false negative cases, respectively. A confidence level threshold equal to 0.4 was set for all the datasets, and in the case of multiple detection results, the one with the highest confidence level was selected. If a model could not meet the confidence level threshold, the detection result was considered *FN* because the test sample images were acquired in advance and could not be re-acquired.

To test whether the optimal model could be used for field identification and assist with plant sampling, the S3–S7 samples were also subjected to PCR detection. These samples were manually identified by experts from the images acquired under condition B (**Table 1**), and were identified by both experienced and inexperienced users using the optimal model from images of T6 and T5 acquired under condition B (**Table 2**), respectively. The DNA was isolated from leaf vein or fruit pith by using the DNAsecure Plant Kit (DP320-03, Tiangen, China) as per the manufacturer’s protocol. The PCR primers used were LAS606 (GGAGAGGTGAGTGGAATTCCGA)/LSS (ACCCAACATCTAGGTAAAAACC). The total volume of the PCR reaction was 25 μL, which consisted of 9.5 μL of ddH_2_O, 12.5 μL of 2 × Taq Master Mix (P112-03, Vazyme, China), 1 μL each of forward and reverse primers (10 μmol/L), and 1 μL of template DNA. PCR amplification was performed in a PCR machine (Bio-Rad T100) using an initial denaturation at 95°C for 3 min followed by 30 cycles at 95°C for 30 s, 58°C for 30 s, and 72°C for 30 s, and then a final extension at 72°C for 5 min. The PCR products were detected by electrophoresis on 1.0% agarose gel, and those with bands the size of the target product (approximately 501 bp) were considered PCR-positive.

### 2.5 Construction of the ‘HLBdetector’

We developed the mobile software ‘HLBdetector’ based on the trained neural network model for HLB disease detection. The application software is available for Android phones and consists of a mobile client and web service application software that supports the acquisition of photos with the phone’s camera or the images stored on the phone. The user can upload the image file to the service interface of a designated server, and the service interface software transfers the received image to the trained neural network model for classification and recognition. The service then provides the classification and recognition result back to the mobile phone, which is displayed on the phone screen.

## 3 Results

### 3.1 Influence of data category on model outcome

The *p*-value, R-value, and F1-score of the Yolov5l-HLB2 model was 3.13%, 5.89%, and 5.56% higher than model Yolov5l-HLB1, respectively, which indicated that including a dataset of HLB-like symptoms (i.e., dataset D) in the training set improved the classification accuracy of the model. Furthermore, compared to Yolov5l-HLB2, Yolov5l-HLB3 had a 0.51% lower *p*-value, a 0.54% higher R-value, and a 0.06% lower F1-score, indicating that including dataset E in the training set was not effective at improving the recognition accuracy of the model (**Table 3**).

**Table 3:** Detection results of three models using different training datasets.

Model	Symptom	TP	FP	FN	P	R	F1
Yolov5l-HLB1 (Dataset A+B+C)	S1	58	4	2	0.9355	0.9667	0.9508
	S2	56	4	4	0.9333	0.9333	0.9333
	S3	67	22	3	0.7528	0.9571	0.8428
	S4	20	2	6	0.9091	0.7692	0.8333
	S5	78	5	2	0.9398	0.9750	0.9571
	S6	24	0	23	1.0000	0.5106	0.6761
	S7	30	5	5	0.8571	0.8571	0.8571
	Average				**0.9039**	**0.8527**	**0.8644**
Yolov5l-HLB2 (Dataset A+B+C+D)	S1	56	5	1	0.9180	0.9825	0.9492
	S2	50	3	6	0.9434	0.8929	0.9174
	S3	62	15	5	0.8052	0.9254	0.8611
	S4	20	1	6	0.9524	0.7692	0.8511
	S5	75	4	1	0.9494	0.9868	0.9677
	S6	31	2	13	0.9394	0.7045	0.8052
	S7	24	4	10	0.8571	0.7059	0.7742
	S8	32	1	0	0.9697	1.0000	0.9846
	S9	32	0	0	1.0000	1.0000	1.0000
	S10	35	2	1	0.9459	0.9722	0.9589
	S11	27	1	0	0.9643	1.0000	0.9818
	S12	44	1	0	0.9778	1.0000	0.9888
	Average				**0.9352**	**0.9116**	**0.9200**
Yolov5l-HLB3 (Dataset A+B+C+D+E)	S1	56	4	1	0.9333	0.9825	0.9573
	S2	47	3	9	0.9400	0.8393	0.8868
	S3	60	16	7	0.7895	0.8955	0.8392
	S4	22	1	4	0.9565	0.8462	0.8980
	S5	76	3	0	0.9620	1.0000	0.9806
	S6	25	2	19	0.9259	0.5682	0.7042
	S7	26	8	8	0.7647	0.7647	0.7647
	S8	32	1	0	0.9697	1.0000	0.9846
	S9	31	0	1	1.0000	0.9688	0.9841
	S10	35	2	1	0.9459	0.9722	0.9589
	S11	27	0	0	1.0000	1.0000	1.0000
	S12	44	3	0	0.9362	1.0000	0.9670
	S13	36	0	0	1.0000	1.0000	1.0000
	S14	35	4	0	0.8974	1.0000	0.9459
	Average				**0.9301**	**0.9170**	**0.9194**

TP, FP, FN, P, R, and F1 indicate true positive, false positive, false negative, precision, recall, and F1-score, respectively. S1, healthy fruit; S2, healthy leaf; S3, blotchy mottling; S4, “red-nose” fruit; S5, zinc-deficiency; S6, vein-yellowing; S7, uniform yellowing; S8, magnesium-deficiency; S9, boron-deficiency; S10, anthracnose; S11, citrus greasy spot; S12, citrus moss.

The values in bold represent the average values for each column.

The recognition accuracy of the three models was further compared for typical and suspected HLB symptoms, including blotchy mottling (S3), “red-nose” fruits (S4), zinc deficiency (S5), vein yellowing (S6), and uniform yellowing (S7). In decreasing order, the corresponding micro F1-scores were Yolov5l-HLB2 (85.19%) > Yolov5l-HLB3 (83.73%) > Yolov5l-HLB1 (83.33%); for *p*-values, the order was Yolov5l-HLB2 (90.07%) > Yolov5l-HLB1 (89.18%) > Yolov5l-HLB3 (87.97%); and for R-values, the order was Yolov5l-HLB2 (81.84%) > Yolov5l-HLB3 (81.49%) > Yolov5l-HLB1 (81.38%). These results suggested that including a dataset of non-HLB symptoms (i.e., dataset E) in the training set did not improve HLB identification accuracy, and Yolov5l-HLB2 outperformed Yolov5l-HLB3. Therefore, Yolov5l-HLB2 was selected as the optimal model.

### 3.2 Influence of test sets acquired under different conditions on model performances

**Figure 5:**
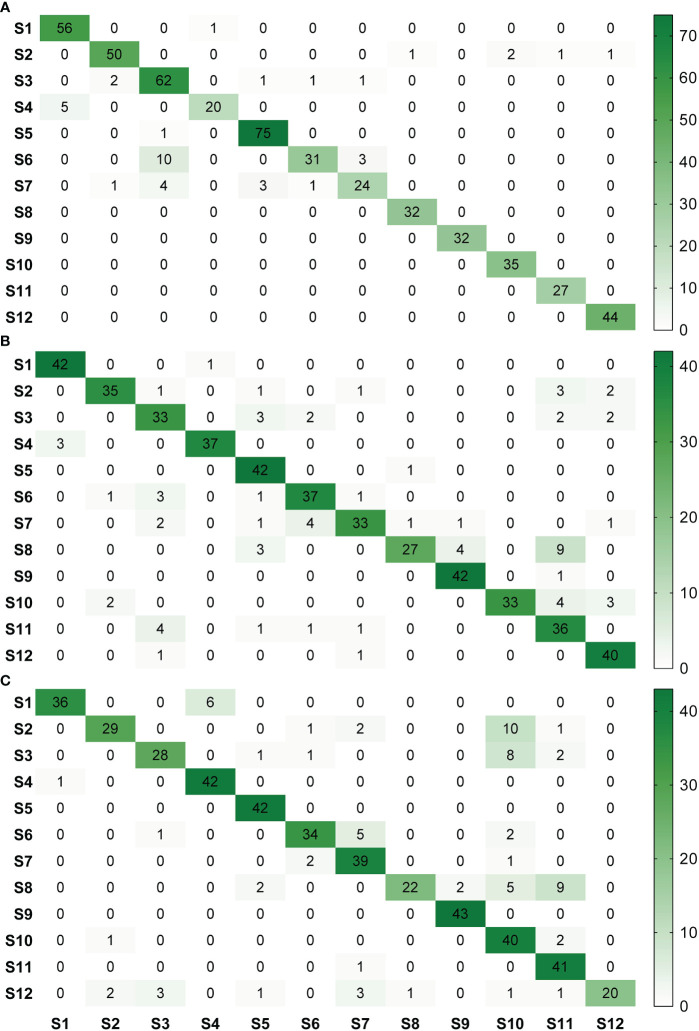
Confusion matrices of model HLB2 for recognising images of 12 citrus diseases acquired under different acquisition conditions. **(A)**, acquisition condition **(A, B)**, acquisition condition **(B, C)**, acquisition condition **(C)**. S1, healthy fruit; S2, healthy leaf; S3, blotchy mottling; S4, “red-nose” fruit; S5, zinc-deficiency; S6, vein-yellowing; S7, uniform yellowing; S8, magnesium-deficiency; S9, boron-deficiency; S10, anthracnose; S11, citrus greasy spot; S12, citrus moss. Note: Missed detection is not reflected in these matrices. The confusion matrices are given in terms of absolute numbers and not percentages.

For image acquisition condition A, 10 samples with vein yellowing (S6) and four samples with uniform yellowing (S7) were misclassified as having blotchy mottling (S3), and five samples with “red-nose” fruits (S4) were misclassified as having healthy fruits (S1) (**Figure 5A**). For image acquisition condition B, four samples with uniform yellowing (S7) were misclassified as having vein yellowing (S6), and four samples with citrus greasy spot (S11) were misclassified as having blotchy mottling (S3). Four samples with magnesium deficiency (S8) were misclassified as having boron-deficiency (S9), and nine samples with magnesium deficiency (S8) and four with anthracnose (S10) were misclassified as having citrus greasy spot (S11) (**Figure 5B**). For image acquisition condition C, six samples with healthy fruits (S1) were misclassified as having “red-nose” fruits (S4), and eight samples of blotchy mottling (S3) and five samples of zinc deficiency (S5) were misclassified as having anthracnose (S10). Five samples with vein yellowing (S6) were misclassified as having uniform yellowing (S7), and nine samples of magnesium deficiency (S8) were misclassified as having citrus greasy spot (S11) (**Figure 5C**). Together, these results indicated the Yolov5l-HLB2 model performed well at recognising 12 symptoms using the images acquired under different conditions.

**Figure 6:**
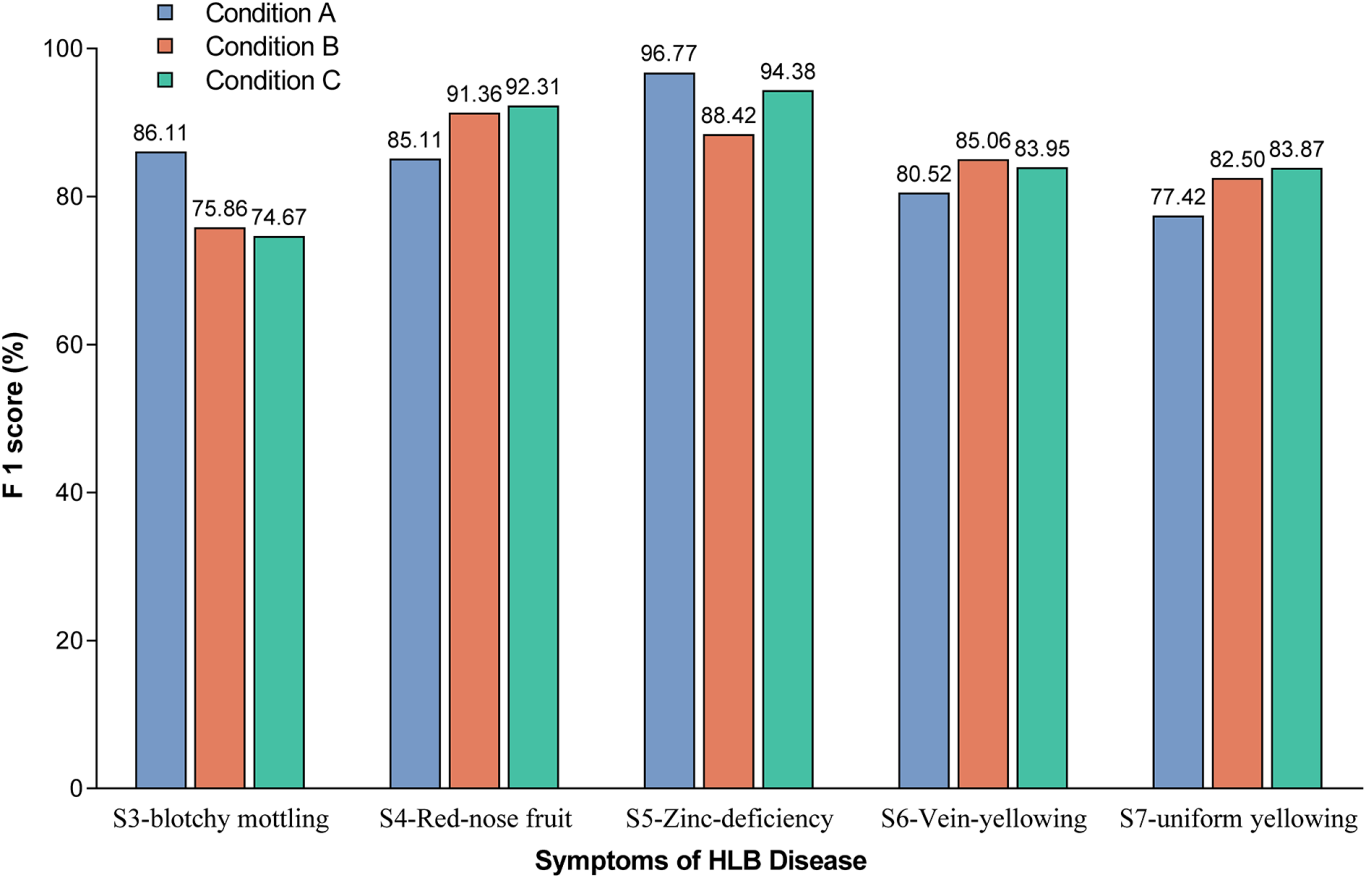
Effect of image acquisition condition on model identification F1-scores for the five symptoms. Note: The value on the bar indicates the exact F1-score.

The F1-scores were further used to evaluate the detection accuracy of the Yolov5l-HLB2 model for the images of two typical HLB symptoms (S3 and S4) and three suspected HLB symptoms (S5, S6, and S7) acquired under different conditions (**Figure 6**). The micro F1-scores of the model for the five symptoms were 85.19%, 84.64%, and 85.84% using image acquisition conditions A, B, and C, respectively. Compared to image acquisition condition A, the following differences were observed: (1) the identification F1-scores of the model for symptom images acquired under condition B were 6.25%, 4.54%, and 5.08% higher for “red-nose” fruits (S4), vein yellowing (S6), and uniform yellowing (S7), respectively, but were 10.25% and 8.35% lower for blotchy mottling (S3) and zinc deficiency (S5), respectively; and (2) the identification F1-scores of the model for symptom images acquired under condition C were 7.20%, 3.43%, and 6.45% higher for S4, S6, and S7, respectively, but were 11.44% and 2.39% lower for S3 and S5, respectively. These results suggest that the Yolov5l-HLB2 model performed better at recognising symptom images S4, S6, and S7 acquired under both conditions B and C than those acquired under condition A, but performed less well on the identification of symptom images S5 and S3.

### 3.3 Comparison of model identification F1-scores between different users

**Figure 7:**
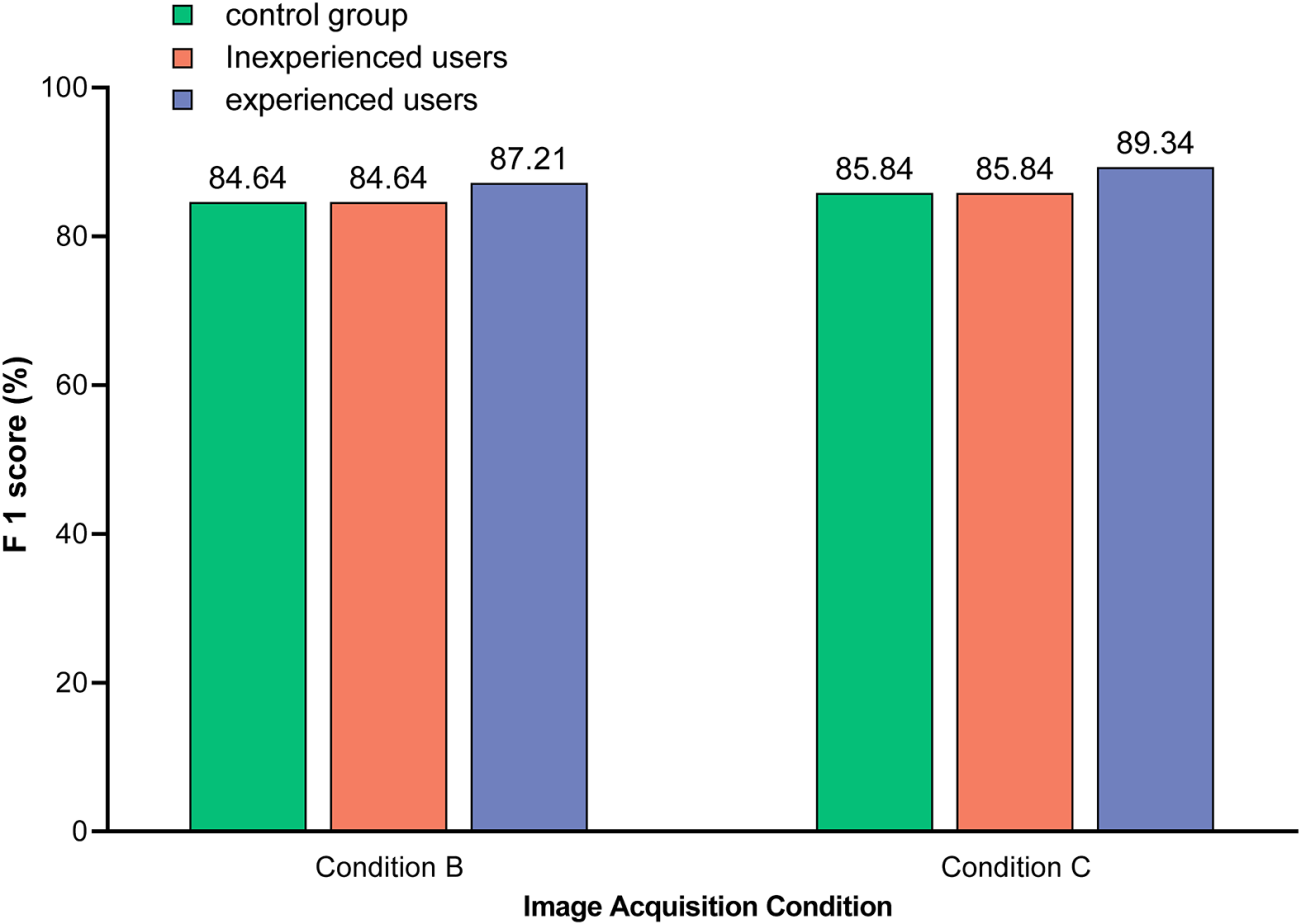
Comparison of the model identification F1-scores between different users. Images used in control group included test sets 4 and 7, which were acquired under condition B and C, respectively, and covered 12 subcategories of symptoms (S1–S12). Images used in unexperienced users included test sets 5 and 8, which were acquired under condition B and C, respectively, and covered all 14 subcategories of symptoms (S1–S14). Images used by experienced users included test sets 6 and 9, which were acquired under condition B and C, respectively, and covered seven subcategories of symptoms (S3–S9). Note: The value on the bar indicates the exact F1-score.

As shown in **Figure 7**, the detection performance of the optimal model was related to the level of user knowledge. Specifically, experienced users were able to initially ignore those images in which symptoms unrelated to HLB were confidently observed, such as anthracnose (S10), citrus greasy spot (S11), citrus moss (S12), sooty mould (S13), and canker (S14). This reduced the risk of HLB symptom misclassification, such as citrus greasy spot (S11) being classified as blotchy mottling (S3), and citrus moss (S12) being classified as blotchy mottling (S3) or uniform yellowing (S7). Therefore, the detection performance of the model was better for experienced users compared to inexperienced users.

### 3.4 PCR validation of citrus HLB disease symptoms recognised using different methods

The PCR-positive rate was higher for blotchy mottling (S3) and “red-nose” fruits (S4) than for the other three symptoms for all three users (**Table 4**). For the same symptom, the higher the user’s experience level, the higher the PCR-positive rate of each symptom except for vein-yellowing (S6) (**Table 4**). The PCR-positive rates of typical HLB symptoms (S3 and S4) detected by the Yolov5l-HLB2 model were considerable, and the PCR-positive rates of other three symptoms detected by this model were close to those classified by experts. This indicates that the optical model can serve as a preliminary screening tool before the field collection of samples for subsequent PCR testing.

**Table 4:** PCR-positive rates of five HLB symptoms classified by different users manually or using model Yolov5l-HLB2.

Symptom	Experienced user(Classified using model)		Inexperienced user(Classified using model)		Expert user(Classified manually)	
	Number of samples	PCR-positive rate (%)	Number of samples	PCR-positive rate (%)	Number of samples	PCR-positive rate (%)
S3	37	86.49	41	78.05	43	90.70
S4	36	91.67	38	86.84	43	97.67
S5	51	47.06	56	42.86	43	53.49
S6	44	38.64	45	37.78	43	30.23
S7	33	21.21	36	19.44	43	27.91
Others	100+301*	3.49	386	3.63	387	0.78
Total/Average (S3–S7)	602	57.01	602	52.99	602	60.00

S3, blotchy mottling; S4, “red-nose” fruit; S5, zinc-deficiency; S6, vein-yellowing; S7, uniform yellowing; Others, symptoms contain in the tested samples except for S3–S7, and samples miss detected. “*” represents the number of images excluded by experienced user manually.

### 3.5 ‘HLBdetector’ development and validation

**Figure 8:**
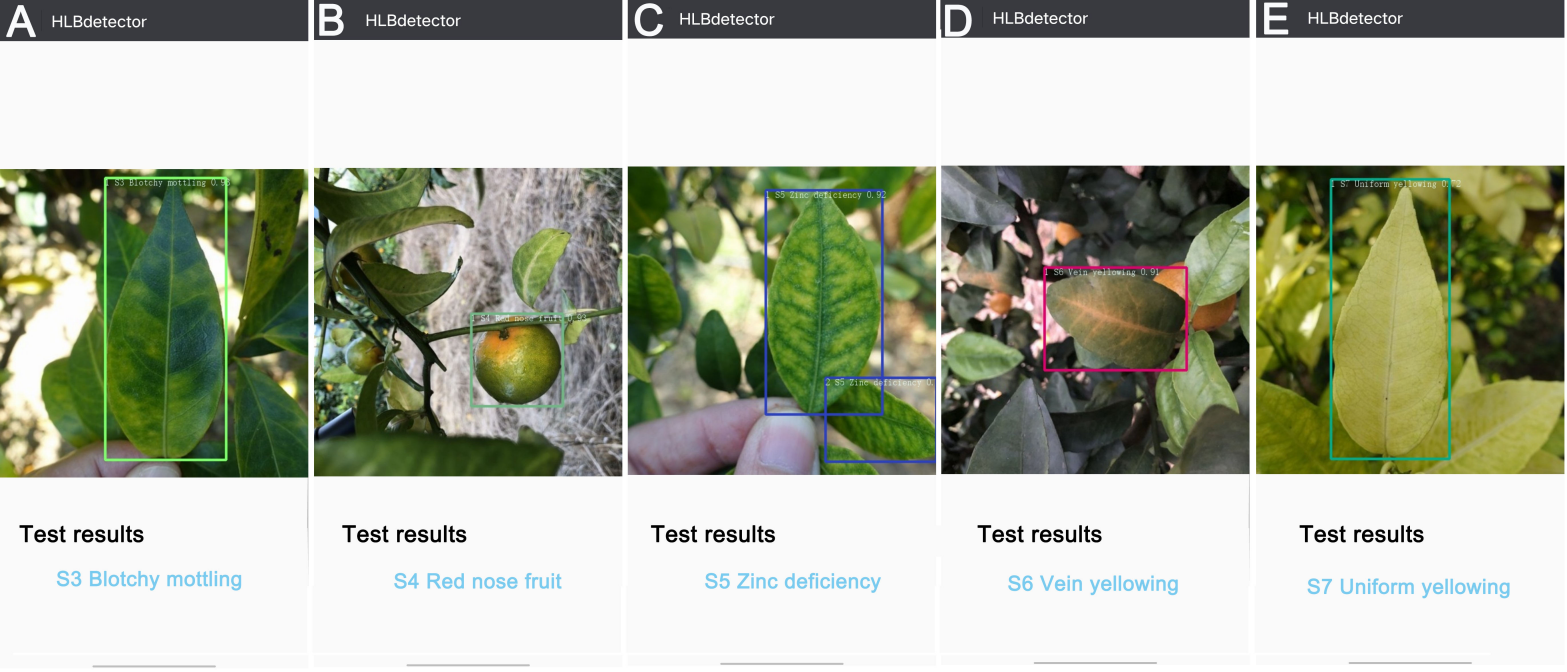
Examples of detection results for five citrus HLB symptoms using ‘HLBdetector’. **(A)**, blotchy mottling (S3); **(B)**, “red-nose” fruits (S4); **(C)**, zinc-deficiency (S5); **(D)**, vein yellowing (S6); **(E)**, uniform yellowing (S7). Note: The colour of hollow squares outside leaves and fruits is randomly selected by the ‘HLBdetector’ software.

The ‘HLBdetector’ employs the Yolov5l-HLB2 model to first obtain a photo from a phone’s album or camera, and then displays a bounding box requesting the user to crop the detection target (e.g., a leaf or fruit) so that it just fits within the box. The image is then scaled down to 640 × 640 pixels and uploaded using an “Identify” button in the application software. The identification result is then shown on the screen. If the confidence level of the identification result is less than 0.4, the user will be prompted to provide a different image. In this study, the photos taken in the field were transferred to an Android phone for testing, and the detection results for the S3 (**Figure 8A**), S4 (**Figure 8B**), S5 (**Figure 8C**), S6 (**Figure 8D**), and S7 (**Figure 8E**) samples were observed on the phone screen. The ‘HLBdetector’ software is also able to correctly detect the location and category of a target object when there are multiple objects in the image (**Figure 8C**).

## 4 Discussion

CNN networks learn the characteristics displayed in a training dataset, and so high accuracy can usually be expected in a model trained using a comprehensive training set (Barbedo, 2018b). In this study, we included the following image datasets groups under five major categories: dataset A included healthy fruits and leaves; dataset B included two typical HLB symptoms (i.e., blotchy mottling leaves and “red-nose” fruits), dataset C included three suspected HLB symptoms that might cause the yellowing of leaves (i.e., zinc deficiency, vein yellowing, and uniform yellowing); dataset D included five HLB-like symptoms of leaves that were not caused by CLas (i.e., magnesium deficiency, boron deficiency, anthracnose, citrus greasy spot, and citrus moss), and dataset E included two HLB-irrelevant symptoms (i.e., sooty mould and canker), respectively. The micro F1-scores of the models trained with datasets A+B+C, A+B+C+D, and A+B+C+D+E when recognising HLB symptoms were 83.33%, 85.19%, and 83.73%, respectively, which indicates that including the dataset containing images of HLB-like symptoms (dataset D) helped improve the accuracy of the model. In contrast, including images of irrelevant symptoms (dataset E) did not improve the accuracy of the resulting model. This is likely because the similar features in dataset D would have helped the CNN network learn the characteristics of the two typical HLB symptoms and the three suspected HLB symptoms, while the features of the two HLB-irrelevant symptoms in dataset E likely differed considerably from the other symptoms and, therefore, were not helpful for the learning process.

Although the Yolov5l-HLB2 model showed good performance when recognising citrus HLB, the model detection accuracy could be further improved. Qi et al. (2022) proposed an improved YOLOv5 model by adding a squeeze-and-excitation module, which showed high accuracy (91.07%) when detecting tomato virus disease. Li et al. (2022a) also proposed an improved YOLOv5-based vegetable disease-detection method, which improves the Cross Stage Partial, Feature Pyramid Networks, and Non-Maximum Suppression modules in YOLOv5s, yielding a mean average precision (mAP) of 93.1%. Furthermore, Li et al. (2022b) employed an improved YOLOv5 model for cucumber disease detection that integrates the Coordinate Attention and Transformer architecture and a multi-scale training strategy and feature fusion network, offering higher detection accuracies and speeds than those obtained from the original YOLOv5 model. Roy et al. (2022) proposed an improved YOLOv4 model for detecting different plant diseases in complex scenarios by including the DenseNet, two new residual blocks, spatial pyramid pooling, and a modified Path Aggregation Network, maximizing both detection accuracy and speed. The previous studies showed that the accuracy of most plant disease-detection models based on YOLO can be improved by modifying the component network modules. Thus, in our future work, we plan to optimise our HLB-detection model to further enhance its detection accuracy.

The backgrounds of images may adversely influence classification results produced by CNN models (Barbedo, 2016; Barbedo, 2018a; Barbedo, 2018b). For example, striking differences have been reported when models trained only with images captured in the field were employed to identify images captured under controlled laboratory conditions and *vice versa* (Ferentinos, 2018). In our study, we used images acquired both in the field and against black or white plates in the laboratory to train our models. Notably, as discussed in Section 3.2, training the models with these two types of images had little impact on the classification results of the five HLB symptoms, yielding a micro F1-score of 85.19% compared to 84.64% for model tested only with field images, and 85.84% for models tested only with laboratory images. This suggests that in the case of HLB detection, the potential influence of image background on detection accuracy may be diluted if a wide variety of image backgrounds are included in initial training sets.

The detection performance of our optimal model was better when used by experienced rather than inexperienced users. In practice, experienced users can take advantage of their knowledge to reduce the inclusion of irrelevant samples in the test set and, thereby, improve the detection performance of the model. In turn, this means that increasing the number of symptoms included in the test sets might increase the chances of misclassification. Moreover, the correct detection rates of the test images with typical and atypical symptoms classified by experts were 91.76% and 81.78% (data not shown), respectively, which indicates that symptom variations might have a serious impact on model detection performance. A possible explanation for this might be that our training dataset was not comprehensive; however, even when training datasets are carefully selected, considering the considerable effort required to capture images and the even more difficult task of labelling the images correctly, it is unpractical to build a training dataset that considers all possible capture conditions and symptom variations (Kamilaris and Prenafeta-Boldú, 2018). It is worth mentioning that such problems will be diminished over time as new images continue to be added to our database.

PCR testing remains the most reliable method for HLB diagnosis, although it is relatively expensive due to skilled operators and specialised equipment required (Li et al., 2007). Compared with the PCR-positive rates for HLB symptoms classified by expert users, our Yolov5l-HLB2 model can be employed as a valuable tool to assist HLB diagnosis, and in particular, can serve as a preliminary screening tool to minimise PCR testing samples. It is worth noting that even the PCR-positive rate of the samples with “red-nose” fruit classified by the inexperienced users was 86.84%, which indicates that the model may serve as a useful recognising tool for this symptom.

Software that can perform automated identification of plant diseases is more useful when it can be used in the field (Ferentinos, 2018). We specifically designed ‘HLBdetector’ to be user-friendly and for field application for the detection of HLB-symptomatic citrus trees. Using this tool has low operational costs and takes only a few seconds using a mobile phone, which has significant advantages for practical agricultural application. In addition, our findings provide an important means for farmers to conduct continuous and timely HLB detection, which facilitates the wider prevention and control of HLB transmission and overcomes the existing problems of citrus HLB detection including the need for manual detection and high PCR testing costs.

## 5 Conclusion

We constructed a dataset containing images of healthy citrus leaves and fruits and those affected by twelve citrus disease symptoms without image augmentation. Using these data, we constructed citrus HLB-detection models using different dataset combinations and the YOLOv5l algorithm. Based on our results, we found that our Yolov5l-HLB2 model outperformed all other models, yielding a micro-F1 score of 85.19%. Our study suggests that similarities between images helped the network learn the characteristic symptomatic features of the HBL, illustrating the opportunities for building image dataset of other plant diseases that could be used for developing disease-detection models. However, our Yolov5l-HLB2 model has some limitations, including the need to further improve detection accuracy. To address this, we plan to optimise our model by modifying the component modules and *via* integration with other algorithms. In addition, we aim to improve the models by obtaining more sample images in future work, for which qPCR instead of PCR will be employed as the gold standard to confirm HLB presence. Nevertheless, we successfully applied the Yolov5l-HLB2 model in the ‘HLBdetector’ app, which can automatically detect HLB symptoms in the field. This user-friendly app can guide citrus fruit growers’ decision-making regarding disease monitoring and intervention to help minimise potential losses.

Overall, our study extends the application prospects of DL in field-based plant-disease diagnosis, providing a reference for the artificial intelligence-based identification of plant diseases and pests and their application. In the future, pilot applications of the ‘HLBdetector’ app will be performed in China to continue enriching our image dataset and optimise the detection model.

## Data availability statement

The raw data supporting the conclusions of this article will be made available by the authors, without undue reservation.

## Author contributions

Conceptualisation, R-ZQ, JZ, and Q-YW; methodology, R-ZQ; validation, R-ZQ, M-XC, R-BW, and TH; data curation, R-ZQ; writing, R-ZQ and S-PC; visualisation, R-ZQ, S-PC, and TH; supervision, JZ, Q-YW, and G-CF. All authors contributed to the article and approved the submitted version.
